# Iodine-131 Dose Dependent Gene Expression in Thyroid Cancers and Corresponding Normal Tissues Following the Chernobyl Accident

**DOI:** 10.1371/journal.pone.0039103

**Published:** 2012-07-25

**Authors:** Michael Abend, Ruth M. Pfeiffer, Christian Ruf, Maureen Hatch, Tetiana I. Bogdanova, Mykola D. Tronko, Armin Riecke, Julia Hartmann, Viktor Meineke, Houda Boukheris, Alice J. Sigurdson, Kiyohiko Mabuchi, Alina V. Brenner

**Affiliations:** 1 Bundeswehr Institute of Radiobiology, Munich, Germany; 2 Biostatistics Branch, Division of Cancer Epidemiology and Genetics, National Cancer Institute, Rockville, Maryland, United States of America; 3 Radiation Epidemiology Branch, Division of Cancer Epidemiology and Genetics, National Cancer Institute, Rockville, Maryland, United States of America; 4 Institute of Endocrinology and Metabolism, Kyiv, Ukraine; 5 NOVA Research Company, Bethesda, Maryland, United States of America; Consiglio Nazionale delle Ricerche (CNR), Italy

## Abstract

The strong and consistent relationship between irradiation at a young age and subsequent thyroid cancer provides an excellent model for studying radiation carcinogenesis in humans. We thus evaluated differential gene expression in thyroid tissue in relation to iodine-131 (I-131) doses received from the Chernobyl accident. Sixty three of 104 papillary thyroid cancers diagnosed between 1998 and 2008 in the Ukrainian-American cohort with individual I-131 thyroid dose estimates had paired RNA specimens from fresh frozen tumor (T) and normal (N) tissue provided by the Chernobyl Tissue Bank and satisfied quality control criteria. We first hybridized 32 randomly allocated RNA specimen pairs (T/N) on 64 whole genome microarrays (Agilent, 4×44 K). Associations of differential gene expression (log_2_(T/N)) with dose were assessed using Kruskall-Wallis and trend tests in linear mixed regression models. While none of the genes withstood correction for the false discovery rate, we selected 75 genes with *a priori* evidence or P kruskall/P trend <0.0005 for validation by qRT-PCR on the remaining 31 RNA specimen pairs (T/N). The qRT-PCR data were analyzed using linear mixed regression models that included radiation dose as a categorical or ordinal variable. Eleven of 75 qRT-PCR assayed genes (*ACVR2A*, *AJAP1*, *CA12*, *CDK12*, *FAM38A*, *GALNT7*, *LMO3*, *MTA1*, *SLC19A1*, *SLC43A3*, *ZNF493*) were confirmed to have a statistically significant differential dose-expression relationship. Our study is among the first to provide direct human data on long term differential gene expression in relation to individual I-131 doses and to identify a set of genes potentially important in radiation carcinogenesis.

## Introduction

One of the most important health consequences of the 1986 Chernobyl nuclear power plant accident is a dramatic increase in thyroid cancer incidence among those who were children or adolescents at the time [1], [2]. Numerous epidemiological studies have established that this is primarily related to iodine-131 (I-131) exposure from the accident to the thyroid gland [3]–[7]. Despite differences in study populations, designs, and dosimetric approaches, reported estimates of relative risk (RR) per unit of absorbed radiation dose in gray (Gy) for those exposed during childhood are remarkably similar among most studies (3–6 per Gy) and compatible with the risks estimated for thyroid cancer from childhood exposure to external radiation [3]–[8].

The study of radiation carcinogenesis in humans can take advantage of instances where relationships are strong and consistent, as in the situation where the thyroid gland is irradiated at a young age. The opportunity for molecular research of radiation-related thyroid cancer is facilitated by the resources of the Chernobyl Tissue Bank (CTB), which systematically collects biological samples from patients with Chernobyl-related thyroid pathology [9]. Earlier post-Chernobyl studies reported genetic differences between radiation-related and sporadic tumors, including specific *RET/PTC* gene rearrangements [10]–[12]. However, subsequent analyses utilizing the CTB samples attributed the associations to the younger age of Chernobyl-exposed patients rather than to their radiation exposure [13], [14]. Recent studies using the CTB materials and high throughput technologies have identified new ‘radiation-specific’ genes [15]–[20]. Perhaps the most compelling finding derives from a report comparing exposed and unexposed papillary thyroid cancer (PTC) cases with young age of onset from Ukraine that found a gain of chromosome band 7q11 based on comparative genomic hybridization, confirmed in independent cases by fluorescence *in situ* hybridization (FISH) and quantitative real-time polymerase chain reaction (qRT-PCR) [20]. However, findings at the gene level are generally inconsistent across studies potentially due to small sample sizes, use of controls from different populations, lack of methodological validation in independent samples, and different analytic approaches. More importantly, none of the previous studies had individual radiation doses assuming that all exposed cases received the same dose. Depending on the true dose-expression relationship, this assumption could cause false positive or false negative associations.

To improve understanding of the molecular consequences of I-131 exposure, we evaluated for the first time differential gene expression in thyroid tissue, defined as a difference in gene expression levels between tumor and corresponding normal thyroid tissue, in relation to individual I-131 thyroid dose estimates. We hypothesized that if dose-related gene expression patterns in tumor tissue truly reflect an important event in radiation carcinogenesis rather than a long-lasting effect of radiation exposure, they should differ from patterns observed in normal tissue; hence our approach of analyzing differential dose-expression relationships in tumor relative to paired normal samples. Our study used RNA specimens from the CTB of patients who underwent thyroid surgery for thyroid cancer in the Ukrainian-American cohort study composed of approximately 13,000 Ukrainian residents <18 years at the time of the accident with individual radioactivity measurements taken within two months after the accident [21].

In the current study, we first conducted an initial screen in half of the cases to identify promising gene candidates that were differentially expressed in tumor and normal tissue specimens in relation to I-131 dose based on whole genome RNA microarrays (phase I). We then validated the top candidates in tumor and normal tissue specimens from the second half of the cases using qRT-PCR (phase II). For the validated candidates we additionally characterized the relationship of gene expression separately in tumor and normal tissue.

## Materials and Methods

### Patients and Tissue Samples

As a result of four sequential screening examinations, 110 prevalent and incident thyroid carcinomas were diagnosed in the Ukrainian-American cohort between 1998 and 2008 at the Laboratory of Morphology of Endocrine System of the Institute of Endocrinology and Metabolism (IEM, Kiev, Ukraine) [3]. The International Pathology Panel, established in the framework of the CTB project, reviewed all pathological diagnoses. To decrease phenotypic heterogeneity of cases, we excluded 5 follicular and 1 medullary thyroid carcinomas resulting in 104 PTCs eligible for analysis; of these 74 (71%) had stored RNA aliquots from tumor and/or normal tissue in the CTB. Cases included and not included in gene expression analyses were similar in many characteristics including I-131 dose and thus our study sample is likely to be unbiased. All tissue samples were taken intraoperatively after patients signed informed consent forms approved by the institutional review boards (IRB) of the IEM and the Coordinating Center of the CTB project (Imperial College Research Ethics Committee, London, UK). Annual review of the entire project was also provided by the IRB of the National Cancer Institute in the USA.

Detailed operating procedures for the collection, documentation, and processing of frozen tumor and normal thyroid tissue samples are available from the CTB website [22] and were developed jointly with the Laboratory of Morphology of Endocrine System of the IEM and the Wales Cancer Bank.

### Dosimetry

Dosimetric methods have been described elsewhere [23]–[25]. Briefly, individual I-131 thyroid doses and their uncertainties were estimated from the combination of thyroid radioactivity measurements, data on dietary and lifestyle habits, and environmental transfer models using a Monte-Carlo procedure with 1,000 realizations per individual [23]. For the analysis, we used the arithmetic mean of each individual’s 1,000 realizations as the best estimate of I-131 dose corrected for thyroid masses typical of the Ukrainian population [3].

### RNA Extraction and Quality Control

Full details of RNA extraction can be obtained from the CTB website [22]. In brief, frozen thyroid tissue is homogenised using a tissue lyser (Qiagen, Hilden, Germany). RNA is extracted using Qiagen column-based systems. RNA is frozen at −80°C in standard 5 µg aliquots in 20 µl. Quality and quantity of isolated total RNA is measured spectrophotometrically (NanoDrop, PeqLab Biotechnology, Erlangen, Germany) and RNA integrity is assessed by the 2100 Agilent Bioanalyser (Life Science Group, Penzberg, Germany). For our analysis, we used only RNA specimens with a ratio A_260_/A_280_ ≥ 2.0 (Nanodrop) and RNA integrity number (RIN) ≥7.5 or ≥5.5 for whole genome microarray and qRT-PCR analyses, respectively (IMGM Laboratories, Martinsried, Germany).

**Figure 1:**
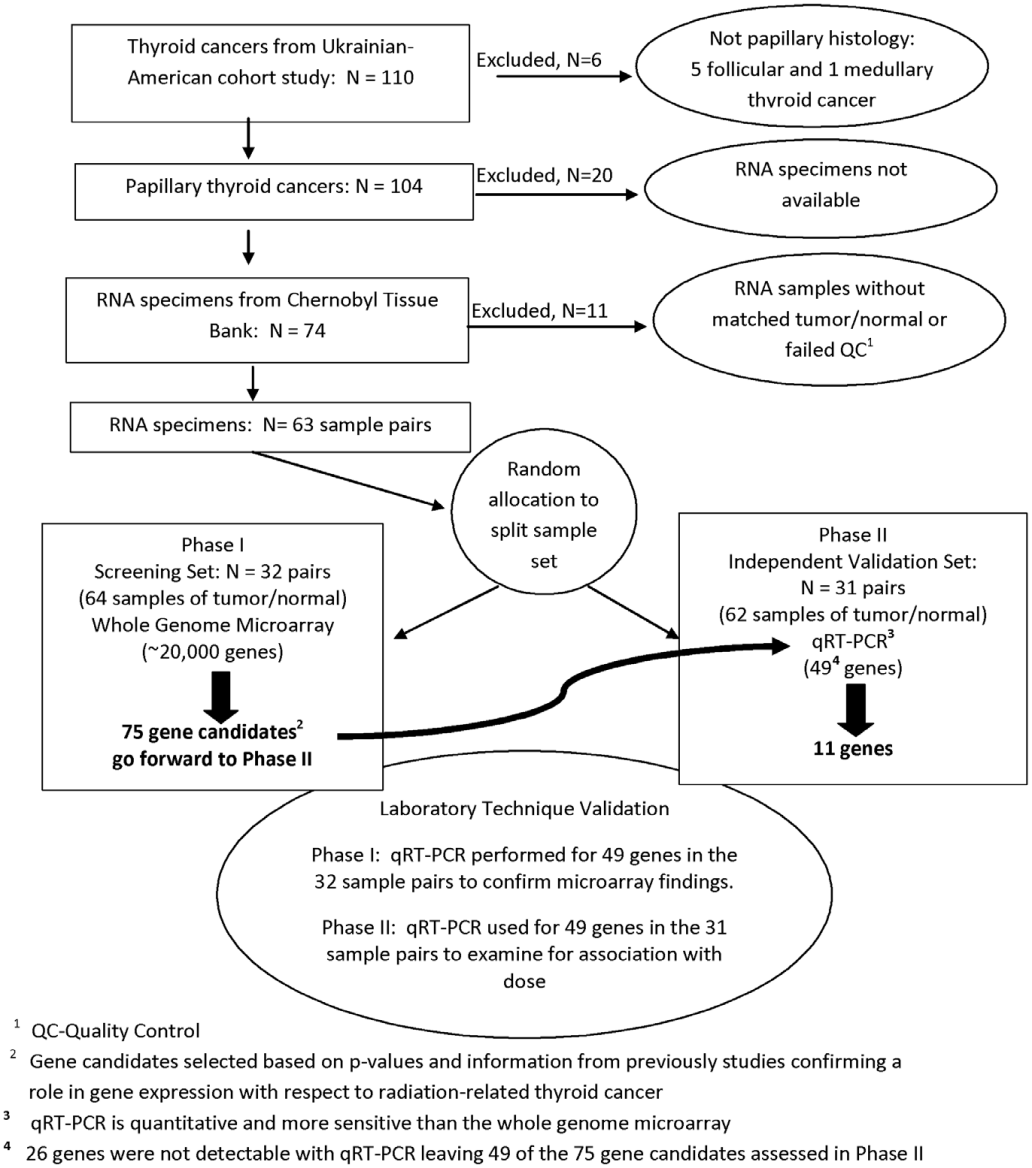
Two stage study design with inclusion and exclusion criteria of cases and selection of gene candidates.

Although the CTB provided 137 RNA specimens for 71 individuals with PTC, we were able to use 126 paired (tumor/normal) RNA specimens corresponding to 63 individuals (Figure 1). Eleven RNA specimens from eight individuals were excluded due to missing complementary tissue (n = 5) or due to failing our quality criteria specified above (n = 6).

### Whole Genome Microarray Analysis (phase I)

Genome wide expression profiling was carried out using the Agilent oligo microarray (4×44 K format) combined with a one-color based hybridization protocol on 64 paired RNA specimens from 32 randomly selected individuals (half the sample set). To assure that individuals selected for microarray gene expression analysis (n = 32) and those remaining for validation analysis by qRT-PCR (n = 31) were similar, we confirmed that their distributions of sex, age, place of residence, and I-131 dose were similar.

Chemicals, kits, and software for the whole genome microarray assay were provided by Agilent Technologies, Waldbronn, Germany. 500 ng of total RNA per specimen (spiked with an internal labeling control) were introduced into an RT-IVT reaction and cDNA was then converted into labeled cRNA by in-vitro transcription step (one color Quick-Amp labeling kit). Quality of labeled non-fragmented cRNA was analyzed (RNA 6000 Nano LabChip kit), and the cRNA was cleaned and quantified (NanoDrop ND-1000 Spetralphotometer). Finally, 1.65 µg of each labeled cRNA sample was fragmented and prepared for one-color based hybridization (gene expression hybridization kit). Hybridization occurred at 65°C over 17 hours on separate microarrays consisting of 41,000 target gene-specific probes (∼20,000 genes) and thousands of control probes. After three washes with increasing stringency, fluorescent signal intensities were detected on the Agilent DNA microarray scanner and extracted from the images using Agilent extraction software. Quantile normalization was applied to the data.

### Phase I: Statistical Analysis of Microarray Data and Selection of Gene Candidates for Independent Validation

We analyzed differential gene expression in tumor compared to normal tissue, obtained by subtracting log_2_ transformed probe signals of the normal tissue (N) from the corresponding tumor tissue (T), log_2_(T)-log_2_(N), in relation to I-131 dose estimates. This approach reduces variability in the gene expression. We used the non-parametric Kruskall-Wallis test (P kruskall) to compare differential gene expression across three dose categories (≤0.30, 0.31–1.0, >1.0 Gy) with cut-off points approximately corresponding to the tertiles of dose distribution among cases, and linear regression models with trend test (P linear) for continuous dose. Only those gene transcripts that had a call “present” in at least 50% of RNA specimens from tumor and normal tissue were included in the analysis of differential gene expression (∼15,000). We corrected for multiple comparisons using the false discovery rate (FDR) [26]. As none of the P values remained significant after FDR correction, we adopted the following criteria to select promising candidates for validation and quantification by qRT-PCR: (P kruskall ≤0.001) or (0.001<P kruskall ≤0.01 and P linear ≤0.01) or (0.01<P kruskall <0.05 and P linear ≤0.001). Overall, 106 candidates out of about 2,500 differentially expressed gene transcripts in relation to dose satisfied the selection criteria. We further narrowed this list to 41 genes that exhibited the lowest P values (P kruskall <0.0005 or P linear <0.0005) and had at least a two-fold difference (increase/decrease) in differential gene expression between highest relative to lowest dose category. An additional 34 genes with P kruskall <0.005 were included in validation because there was evidence in the literature for gene amplification (copy number alteration, CNA >3) or strong up−/down-regulation in other studies of thyroid cancer in irradiated populations [19], [27]. Thus, 75 genes were selected for validation by means of qRT-PCR (Table S1). In addition, to evaluate agreement between whole genome microarray and qRT-PCR measurements, we ran qRT-PCR (Applied Biosystems, Darmstadt, Germany) for these genes among the 32 individuals included in Phase I.

### Phase II: Quantitative RT-PCR Analysis

To validate our microarray findings, we evaluated gene expression by qRT-PCR (TaqMan primer probe assays) on 62 paired RNA specimens from the remaining 31 individuals. All chemicals for qRT-PCR using TaqMan chemistry were provided by Applied Biosystems, Darmstadt, Germany. Due to technical reasons individual primer probe assays were either run on a 96-well qRT-PCR platform or using another platform called low density array (LDA). Twenty four of the 75 genes were measured in duplicates, because of available space on the platforms and in order to improve the statistic. A 0.75 µg RNA aliquot of each individual was reverse transcribed using a two-step PCR protocol (High Capacity Kit). 50 µl cDNA (equivalent to about 0.25 µg RNA) was mixed with 50 µl 2 x RT-PCR master mix and pipetted into 2 of 8 fill ports of the LDA. Cards were centrifuged twice (1,200 rpm, 1 min, Multifuge3S-R, Heraeus, Germany), sealed, and transferred into the 7900 qRT-PCR instrument. The qRT-PCR was run for two hours following the qRT-PCR protocol for 384-well LDA format. Taqman chemistry for the 96-well platform was used similarly except the volume per reaction was adjusted to 20 µl. All technical procedures for qRT-PCR were performed in accordance with standard operating procedures implemented in our laboratory in 2008 when the Bundeswehr Institute of Radiobiology became accredited according to DIN EN ISO 9001/2008.

We ran four RNA specimens in triplicate on three different LDAs to establish an upper limit of the linear-dynamic range of the threshold cycles (CT). The upper limit of the CT was 30, so we used only CT values <30 for analysis. CT values were normalized relative to the median gene expression of the examined genes. This approach proved to be more robust compared to the use of 18S rRNA duplicates spotted on each LDA for normalization purposes. The mean coefficient of variation (CV) of qRT-PCR measurements was <2.5% and 95% of triplicate measurements had CV <4% (Figure S1). Differential gene expression reflecting fold-change differences of RNA copy number in tumor tissue relative to the corresponding normal tissue (used as calibrator) was calculated by subtracting the associated CT values (delta-delta-CT-approach).

### Reliability and Comparison of Results by Different Techniques in Phase I and Phase II

Both methods measured on 32 individuals included in phase I revealed comparable results in 70.6% (Figure S2A). Mean differential gene expression from whole genome microarray examined on phase I individuals was also highly correlated with qRT-PCR measurements examined on phase II individuals (comparison of different methods on different individuals, r^2^ = 0.81, Figure S2B). Finally, qRT-PCR measurements of individuals included and not included in phase I were also highly correlated (r^2^ = 0.98, Figure S3), supporting reliability of the methods.

### Phase II: Statistical Analysis of qRT-PCR

To confirm phase I findings, the phase II analyses used only individuals (n = 31) not included in phase I. After a power or log transformation and/or removal of outliers for selected genes, normalized CT values of all genes were normally distributed. We first computed residuals from standard linear models fitted to gene expression values *y* adjusting for age at thyroid surgery (3 categories), sex, and oblast or state of residence (Chernigov, Zhytomyr, Kiev),
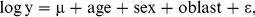
(1)where µ is the overall mean expression level. We then assessed the relationship between I-131 dose and gene expression in linear mixed models with individual specimens as the outcome variable. These models account for correlations of the tumor and normal tissue measurements taken on the same individual and also accommodate duplicate measurements on the same person for the same tissue type.

The models were
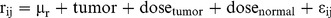
(2)where r_ij_ denotes the residual from model (1) for subjects i (i = 1, 2,…,31) on the j^th^ sample (j = 1, 2 for tumor and normal tissue), and ε_ij_ is normally distributed error term that also incorporates correlations from repeated measurements on the same sample. The dose effect in tumor samples is quantified by dose_tumor,_ and the dose effect in normal tissue is given by dose_normal._ To assess differences in dose effect by tissue type, we tested the null hypothesis H_0_: dose_tumor_  =  dose_normal._ Again, I-131 dose was used in two ways: in 3 independent dose groups, which leads to a 2 degree of freedom Wald test P value for H_0_, and using the 3 ordered dose groups, corresponding to a 1 degree of freedom test for H_0_.The plots show the residuals and fitted dose slopes for normal and tumor tissue. Fold changes in expression associated with dose were computed as two to the power of the difference in the slopes, i.e. 2ˆ(dose_tumor_ - dose_normal_). Parameters for the mixed models were estimated using the restricted maximum likelihood method incorporated in PROC MIXED, SAS 9.1.3 [28].

## Results

### Characteristics of PTC Cases

Of 63 cases in our study, 56% were female and 54% were residents of the Chernigov oblast (Table 1). Age at time of the accident ranged from 0 to <18 years (mean 7.9 years) and cancers were diagnosed 12.5–21.6 years after the accident (mean 16.5 years). Mean I-131 thyroid dose was 1.25 Gy, ranging between 0.008 and 8.6 Gy. Means for the 3 dose categories were 0.11, 0.57, and 2.62 Gy, respectively. The most common histological subtype of PTC was mixed (48%) and the remainder consisted of follicular (25%), classic papillary (19%), and solid (8%) subtypes. The mean of the largest tumor diameter was 16.0 mm, with a range from 6.0 to 45.0 mm.

**Table 1:** Characteristics of papillary thyroid cancer cases from the Ukrainian-American study cohort, 1998–2008.

Characteristic	N or Mean ± SD	% or range
Gender, female	35	56
Oblast of residence^1^		
Zhytomyr	15	24
Kiev	14	22
Chernigov	34	54
Age at exposure, year^2^	7.9±4.6	0−<18
Latency, year^3^	16.5±2.7	12.5–21.6
I-131 thyroid dose, Gy	1.25±1.68	0.008–8.6
0.008–0.30	23	36
0.31–1.0	23	36
1.1–8.6	27	43
Tumor size, mm^4^	16.0±7.8	6.0–45.0
Histological subtype of PTC		
solid	5	8
papillary	12	19
follicular	16	25
mixed	30	48

1 At the time of first screening examination.

2 On April 26, 1986.

3 Differences between surgery date and April 26, 1986.

4 Largest dimension at pathomorphology.

NB: Not all percentages sum to 100 due to rounding.

### Whole Genome Microarray

Of 19,596 gene mRNAs (41,079 transcripts) spotted on the whole genome microarray, on average 73.4% (range: 63.3%–91.0%) were distinguishable from background (expressed). The total number of gene transcripts differentially expressed in relation to I-131 dose was 2,500; of these we selected 75 gene candidates for validation by qRT-PCR (Table S1).

### qRT-PCR

**Figure 2:**
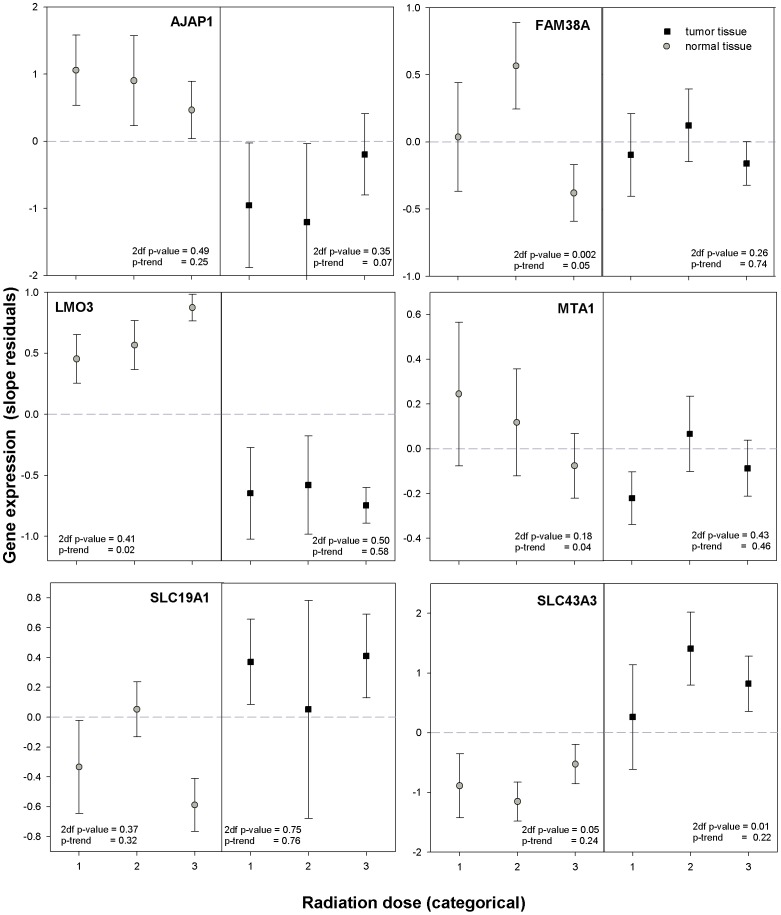
Residual gene expression by tissue type in relation to I-131 thyroid dose estimates. Note: Mean of residual gene expression after removing the effects of age, oblast, and sex is plotted separately for normal tissue (left part of the graph) and tumor tissue (right part of the graph) against the mean of three I-131 dose categories (0.11, 0.57, 2.62 Gy). Circles with grey fills correspond to mean gene expression values for normal tissue and squares with black fills correspond to mean gene expression values for tumor tissue. Error bars represent 95% confidence intervals. P-values for association with dose are based on a 2 degree of freedom test and a 1 degree of freedom trend test, respectively, and given separately for normal and tumor tissue in the bottom of each panel.

**Figure 3:**
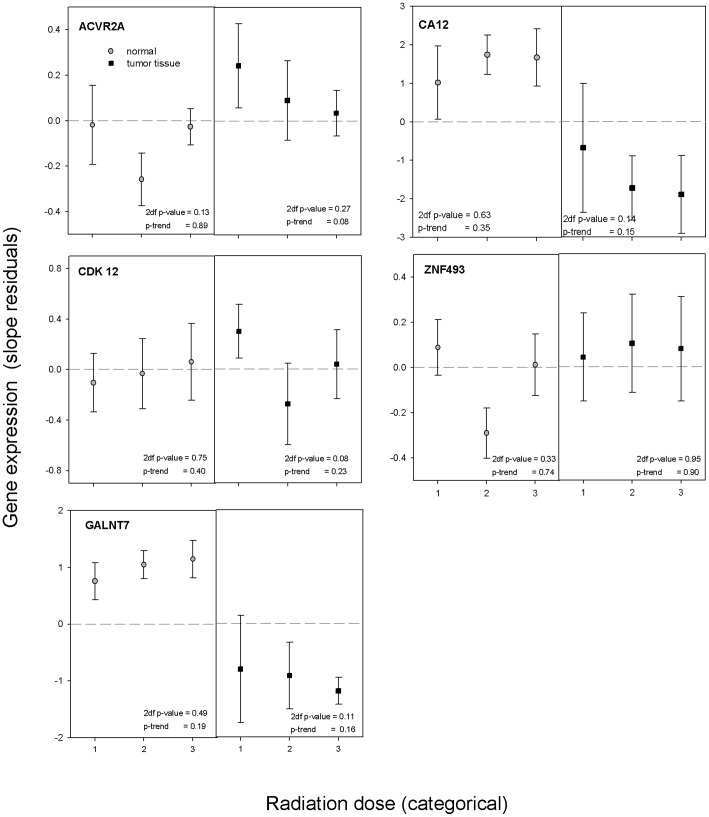
Residual gene expression by tissue type in relation to I-131 thyroid dose estimates. Note: Mean of residual gene expression after removing the effects of age, oblast, and sex is plotted separately for normal tissue (left part of the graph) and tumor tissue (right part of the graph) against the mean of three I-131 dose categories (0.11, 0.57, 2.62 Gy). Circles with grey fills correspond to mean gene expression values for normal tissue and squares with black fills correspond to mean gene expression values for tumor tissue. Error bars represent 95% confidence intervals. P-values for association with dose are based on a 2 degree of freedom test and a 1 degree of freedom trend test, respectively, and given separately for normal and tumor tissue in the bottom of each panel.

Of 75 genes assayed, 15 developed no amplification plots, 5 yielded results in <10 individuals, and 6 exhibited high variability probably caused by an insufficient primer probe design making these 26 genes uninformative. For 11 of the remaining 49 genes, the dose-related expression in tumor tissue was statistically significantly different from the dose-related expression in normal tissue when dose was used as a categorical or ordinal variable in the linear mixed models (Table 2). Associations significant for both categorical and ordinal dose were found for the following 6 genes: *ACVR2A*, *CA12*, *CDK12*, *FAM38A*, *LMO3*, *MTA1* (Table 2). Three genes (*SLC19A1*, *SLC43A3*, *ZNF493*) had statistically significant differences in differential gene expression for categorical dose (2 degree of freedom tests), but not for ordinal dose, suggesting non-linear dose-expression relationships; and two genes (*AJAP1*, *GALNT7*) had a statistically significant difference in dose-expression relationship for ordinal dose, but not for categorical dose. For *SLC43A3* the associations between gene expression and dose were restricted to tumor tissue (2 degree of freedom test) and for *FAM38A (*2 degree of freedom and 1 degree of freedom tests), *SLC43A3* (2 degree of freedom test), *LMO3* (1 degree of freedom test), *MTA1* (1 degree of freedom test) to normal tissue (Figures 2 and 3). For *CA12*, *GALNT7*, *LMO3*, and *SLC43A3* there is good separation in expression between tumor and normal tissue at each dose level as well as a suggestion of opposing trends with dose. However, in most instances, significant differences in dose-response by tissue type appeared to be due to the heterogeneity of dose-response patterns in the absence of a monotone dose-response in either tissue type.

**Table 2:** Summary statistics for genes with significant differential dose-expression relationship based on qRT-PCR measurements.

				fold-change per dose category^3^			
Gene	Cytoband	CNV^1^	N^2^	1	2	P^4,6^	P trend^5,6^
*ACVR2A*	2q22.3	no	31	1.1	0.9	0.001	0.02
*AJAP1*	1p36.32	amplified	20	1.1	2.4	0.06	0.03
*CA12*	15q22.2	no	31	0.3	0.3	0.01	0.02
*CDK12*	17q12	no	31	0.7	0.7	0.01	0.04
*FAM38A*	16q24.3	amplified	31	0.8	1.3	0.0004	0.04
*GALNT7*	4q31.1	no	31	0.8	0.6	0.06	0.02
*LMO3*	12p12.3	amplified	31	0.9	0.7	0.01	0.01
*MTA1*	14q32.33	amplified	31	1.3	1.4	0.01	0.002
*SLC19A1*	21q22.3	amplified	31	0.7	1.2	0.002	0.13
*SLC43A3*	11q12.1	amplified	31	2.5	1.1	0.01	0.99
*ZNF493*	19p12	no	31	1.3	1.0	0.0001	0.74

1 CNV, Copy number variation as reported in Stein et al. with either amplified regions or not (no) is shown for each cytoband where our candidate genes are located.

2 Number of paired (tumor/normal tissue) observations.

3 Columns with subtitles 1 and 2 refer to dose categories (1 and 2) and reflect the fold change in differential gene expression for a specific dose category relative to the referent dose category (0). Fold change in expression associated with dose were computed as two to the power of the difference in the slopes, i.e. 2ˆ(dose_tumor_−dose_normal_).

4 Two degree of freedom test in differential dose-response.

5 One degree of freedom trend test in differential dose-response.

6 All models of differential dose-response were adjusted for tissue type, attained age, sex, and oblast of residence.

## Discussion

Because the relationship between irradiation at a young age and risk of thyroid cancer is strong and strikingly consistent, this tumor provides an excellent model for studying radiation carcinogenesis in humans. Here, we employed measurement-based individual I-131 doses estimated for a cohort of Ukrainian residents who were <18 at the time of the Chernobyl accident and RNA specimens from fresh frozen thyroid tissue provided by the CTB. For the first time, we conducted analyses of dose-dependent gene expression in papillary thyroid carcinoma relative to normal thyroid tissue from the same individuals across the entire genome and confirmed findings for 11 genes by qRT-PCR in RNA specimens from a separate set of cases.

Several points should be considered when comparing our results to prior studies. Transcriptional profiling using microarrays that allows simultaneous evaluation of thousands of gene transcripts has been extensively used to gain insights into the molecular changes induced by ionizing radiation [29]–[35]. However, it was primarily applied to various cell types *in vitro*: cell cultures of human keratinocytes [31], lymphoblastoid cells [34], etc. A few studies including animal models or patients treated with radiotherapy investigated gene expression soon after *in vivo* irradiation [32], [33], [35]. The genes that exhibited dose-related changes following exposure to ionizing radiation in these studies are most likely to be useful as early biological endpoints and/or biomarkers of exposure. Their usefulness in terms of long-term cancer risk in humans remains to be established. There were also some mechanistic studies that compared gene expression in radiation-related cancers with gene expression in sporadic cancers or corresponding normal tissue [15]–[20], but none of these involved individual dose estimates. Our study has attempted to bridge this gap. Using individual I-131 dose estimates, we evaluated dose in three categories, without assumptions about the dose-response shape and assuming a linear dose-response. Because different radiobiological mechanisms might occur with increasing radiation doses including cell killing and induction of persistent chromosomal rearrangements, which themselves are known to follow non-linear relationship [36], [37], a curvilinear relationship for differential gene expression is plausible. Consistent with this idea, at least some genes (*SLC19A1*, *SLC43A3*, *ZNF493*) exhibited a suggestion of non-linearity in differential dose-response.

The 11 genes that were validated in an independent case set by qRT-PCR in our study (Table S2) are all located on different chromosomes and belong to different biological pathways, including cell adhesion (*AJAP1*, *FAM38A*), energy metabolism (*CA12*), transcription or DNA methylation (*LMO3*, *ZNF493*, *MTA1*, *SLC19A1*), and growth/differentiation (*CDK12*, *ACVR2A*). All these pathways have been implicated in cellular responses to ionizing radiation [31], [34]. More importantly, four specific genes (*CA12*, *GALNT7*, *LMO3*, and *SLC43A3)* were previously reported by Stein et al. to be uniquely deregulated in post-Chernobyl thyroid cancers [19] and one gene (*FAM38A*) was previously identified by Ory et al. in a study of thyroid cancer following irradiation for a first primary cancer in childhood [27]. In addition to finding these five genes differentially expressed in an independent set of irradiated cases, we provided evidence that their differential expression appears to be dose-dependent. Therefore, these genes are particularly attractive candidates for further validation studies focusing on mechanisms of radiation carcinogenesis. None of the promising genes identified in our study was located in 7q11.22–11.23; gain in this chromosomal region was reported in young-onset post-Chernobyl PTCs [20].

Several clues emerged from our study that could guide future studies of radiation carcinogenesis. Dose-dependency for differential gene expression detected years after exposure likely represents a late and/or long lasting effect of radiation. One mechanism by which radiation-induced changes could be sustained over time is through inheritance of DNA damage [38]. It has been reported that ionizing radiation is able to induce a specific type of DNA damage, notably copy number alterations (CNAs) [39]. Numerous studies of sporadic cancers have established that CNAs can shape the tissue transcriptomes and influence gene expression [40]. Therefore, if radiation-induced CNAs are maintained during tumorigenesis, dose-related differential gene expression might be partially attributed to dose-related changes in CNAs. This idea finds some support in studies that reported overlap in CNAs and genes uniquely deregulated in post-Chernobyl tumors [19] and significant overexpression of one gene among several examined within the gained 7q11.22–7q11.23 region [20]. Another emerging mechanism through which radiation may induce and perpetuate long-lasting changes in gene expression is epigenetics [41], [42]. Epigenetic changes indirectly affect DNA by altering DNA methylation, chromatin remodeling, and microRNA (miRNA) expression rather than DNA structure. Our finding of dose-dependent expression of certain genes (*FAM38A*, *SLC43A3*, *LMO3*, *MTA1*) in normal thyroid tissue may be related to radiation-induced epigenetic changes. Furthermore, dose-related expression changes in normal tissue might offer an additional opportunity to assess individual susceptibility to radiation exposure [43], [44]. Simultaneous evaluation of gene expression, CNAs, and epigenetic changes might provide clues to the complex effects of ionizing radiation and radiation carcinogenesis.

Our study has several unique strengths. First, we used individual I-131 dose estimates based on radioactivity measurements taken shortly after the accident [3], [6]. Second, cases arose within a well-characterized cohort screened for thyroid cancer according to a standardized protocol and irrespective of dose, minimizing the impact of unmeasured confounding. While the total number of cases with paired RNA samples (n = 63) used for whole genome microarray analysis (Phase I, n = 32) and qRT-PCR (Phase II, n = 31) is larger than in previous studies of irradiated populations, collectively these represent 60% of all PTC cases diagnosed in the Ukrainian-American cohort due to screening. However, cases included and not included in gene expression analyses were similar in many characteristics, including dose, and thus our sample is likely to be unbiased. Comparison of gene expression measurements performed by whole genome microarrays (phase I) and qRT-PCR (phase II) on the same individuals together with evaluation of methodological variability of qRT-PCR is all in line with previous work [45], [46] and lessens concern that our findings are due to methodological artifacts.

There are also several limitations to be borne in mind when interpreting our results. We did not take into account of the impact of uncertainties in dose estimates, 95% of which are typically attributable to unknown thyroid gland mass and I-131 content in the thyroid gland in 1986 [47]. However, these dose estimates compare favorably to other studies of environmentally exposed populations that exclusively relied on retrospective dose reconstruction and did not have individual measurements of radioactivity. Moreover, given the source and type of errors in the dose estimates [47], these likely resulted in underestimation of the true gene expression relationship. Therefore, the top genes are unlikely to be false positive findings. Our ability to accurately quantify the magnitude of dose-response for differential gene expression and to characterize its shape was limited due to the small sample size. As this analysis is exploratory, we also did not formally adjust for multiple testing in the selection of our genes and the replication. Validation of our findings by independent methods and in independent cases including radiation-related thyroid cancers with high quality dose estimates and in sporadic thyroid cancers is important. Future examinations should focus on the evaluation of changes in 11 gene candidates at the DNA level (DNA sequencing, methylation-status), the posttranscriptional level (miRNA) and the translational level (protein expression, e.g. immunohistochemistry).

In summary, our study is among the first to provide direct human data on long term gene expression in relation to measurement-based individual I-131 doses. By studying PTCs arising after the Chernobyl accident, we identified 11 genes that exhibited evidence of dose-dependent expression in cancerous relative to normal thyroid tissue and, therefore, potentially important in radiation carcinogenesis. Our study also serves as a basis for further dose-dependent studies of gene expression, CNAs, and epigenetic changes and their role in radiation susceptibility and carcinogenesis.

## Supporting Information

Figure S1 **Methodological variability of qRT-PCR measurements (n = 47) performed on 4 RNA samples (RNA_1–4) in three independent experiments.** Short dashed lines show mean coefficient of variation (CV) of threshold cycle (CT)-values and box plot whiskers reflect 10% and 90% percentiles. All outliers are shown.
(TIF)

Figure S2 **Comparison of whole genome microarray data (log2 transformed) with corresponding qRT-PCR measurements (log2 transformed).** Measurements using both methods were performed either on the same individuals (n = 32) from phase I (A) or on different individuals (B) originating from phase I (n = 32) and phase II (n = 31).Vertical and horizontal lines in (A) reflect two-fold changes in differential gene expression, considered to represent control values due to methodological variation. Areas highlighted in grey depict comparable results as measured by both methods, e.g. genes being up regulated (up/up), control (middle part) or down regulated (down/down). Areas called ‘false positive’ or ‘false negative’ depict > two-fold differential gene expression by microarray data given control values of qRT-PCR measurements. Areas called ‘inverse’ reflect opposing gene expression measurements for the two methods. Results of these comparisons are summarized in the inserted table (lower right corner of panel A). Error bars in (B) represent the standard error of the mean.
(TIF)

Figure S3 **Comparison of qRT-PCR measurements (log2 transformed mean differential gene expression) performed on different individuals from phase I (n = 32) and phase II (n = 31).** Error bars represent the standard error of mean.
(TIF)

Table S1 **Complete list of 75 genes selected for validation by qRT-PCR based on analysis of differential dose-expression relationship of microarray data and/or evidence from previous studies.** Note: ^1^Non-parametric Kruskall-Wallis test for differential gene expression across three dose categories (≤0.30, 0.31–1.0, >1.0 Gy). ^2^Linear trend test for differential gene expression with continuous dose. ^3^Estimate of linear dose-response slope based on continuous dose.
(DOC)

Table S2 **Summary information for 11 genes with significant differential dose-expression relationship based on qRT-PCR measurements.** Note: ^1^Gene description and mechanism of action as defined in National Center for Biotechnology Information (NCBI) Entrez Gene, June 2011. ^2^Based on review of Gene References Into Functions (RIF) and NCBI bibliography as well as additional literature searches in PubMed focused on reports of thyroid cancer or cancer-related biological processes. Abbreviations: TGF-beta, transforming growth factor-beta; PTC, papillary thyroid cancer; ALL, acute lymphoblastic anemia.
(DOC)
