# Supplementary material for: Iodine-131 Dose Dependent Gene Expression in Thyroid Cancers and Corresponding Normal Tissues Following the Chernobyl Accident
Source: PLoS One. 2012 Jul 25;7(7):e39103. doi: 10.1371/journal.pone.0039103 (PMC3405097; doi:10.1371/journal.pone.0039103)
Supplement: Table S1 — Complete list of 75 genes selected for validation by qRT-PCR based on analysis of differential dose-expression relationship of microarray data and/or evidence from previous studies. Note: 1Non-parametric Kruskall-Wallis test for differential gene expression across three dose categories (≤0.30, 0.31–1.0, >1.0 Gy). 2Linear trend test for differential gene expression with continuous dose. 3Estimate of linear dose-response slope based on continuous dose. (DOC) [file pone.0039103.s004.doc]

**Supplemental Table 1.** Complete list of 75 genes selected for validation by qRT-PCR based on analysis of differential dose-expression relationship of microarray data and/or evidence from previous studies.

| **Probe ID** | **Gene Symbol** | **Cytoband** | **P kruskal^1^** | **P linear^2^** | **Slope^3^** |
| --- | --- | --- | --- | --- | --- |
| A_24_P255123 | A_24_P255123 | 22q11.23 | 0.008311 | <0.000001 | -0.62515 |
| A_24_P315581 | A_24_P315581 | 4p16.1 | 0.020633 | 0.00045 | 0.65355 |
| A_24_P752279 | A_24_P752279 | 16p13.3 | 0.000618 | 0.33289 | 0.39824 |
| A_24_P845649 | A_24_P845649 | 16p13.3 | 0.00181092 | 0.375002491 | 0.029741732 |
| A_32_P185089 | A_32_P185089 | 1p36.32 | 0.004563442 | 0.458294069 | 0.016689106 |
| A_32_P90468 | A_32_P90468 | 17q24.3 | 0.002637 | 0.00048 | -0.3725 |
| A_24_P366122 | ACBD4 | 17q21.31 | 0.000895 | 0.14403 | 0.09391 |
| A_23_P153930 | ACVR2A | 2q23.1 | 0.043679 | <0.000001 | -0.52968 |
| A_24_P711809 | AI825645 | 9q34.3 | 0.002167953 | 0.053503677 | 0.089442917 |
| A_24_P264611 | AK092264 | 16q24.3 | 0.004896554 | 0.095492793 | 0.040393558 |
| A_32_P735636 | AK095260 | 13q34 | 0.004896091 | 0.36169136 | 0.181543729 |
| A_24_P306034 | ANKDD1A | 15q22.31 | 0.005798 | 0.00001 | 0.64796 |
| A_32_P10683 | AW389851 | 16q24.3 | 0.000203 | 0.06982 | 0.3094 |
| A_24_P922684 | BC020539 | 5q31.1 | 0.026238 | 0.00018 | -0.13603 |
| A_24_P740549 | BC038431 | 3q25.2 | 0.014632 | 0.00007 | -0.23073 |
| A_32_P199506 | BU191598 | 21q22.3 | 0.000073 | 0.05354 | 0.34495 |
| A_24_P941038 | C10orf72 | 10q11.22 | 0.19322 | 0.00165 | 0.2244 |
| A_23_P344515 | C16orf3 | 16q24.3 | 0.007446583 | 0.287324094 | 0.187241483 |
| A_24_P104926 | C20orf144 | 20q11.22 | 0.005313524 | 0.448183197 | 0.051170936 |
| A_23_P132139 | C21orf58 | 21q22.3 | 0.001282312 | 0.268696109 | 0.095177566 |
| A_24_P330518 | CA12 | 15q22.2 | 0.08234 | 0.00003 | 0.39346 |
| A_23_P84849 | CACNA1B | 9q34.3 | 0.00325502 | 0.348016651 | 0.016921189 |
| A_32_P155645 | CCDC112 | 5q22.3 | 0.042785 | <0.000001 | -0.56785 |
| A_32_P27046 | CHGA | 14q32.12 | 0.006657 | <0.000001 | 0.42745 |
| A_23_P44586 | COX5A | 15q24.1 | 0.502117836 | 0.004826386 | -0.078499906 |
|  |  |  |  |  |  |
| **Probe ID** | **Gene Symbol** | **Cytoband** | **P kruskal^1^** | **P linear^2^** | **Slope^3^** |
| A_23_P8900 | COX6C | 8q22.2 | 0.031640593 | 0.000644278 | -0.055873345 |
| A_23_P159650 | COX7B | Xq21.1 | 0.157430854 | 0.00000194 | -0.113374595 |
| A_23_P159119 | CRKRS | 17q12 | 0.020101 | 0.00097 | -0.48499 |
| A_23_P24433 | CTSF | 11q13.1 | 0.621237597 | 0.000603787 | 0.131333123 |
| A_24_P242036 | dJ222E13.2 | 22q13.2 | 0.001172322 | 0.146357879 | 0.205949093 |
| A_32_P160254 | DKFZP434I0714 | 4q31.3 | 0.004358 | 0.00009 | -0.163 |
| A_23_P106559 | ENST00000074056 | 16p13.3 | 0.000664 | 0.5412 | 0.00367 |
| A_24_P169073 | FAM131C | 1p36.13 | 0.001912441 | 0.203036201 | 0.244931583 |
| A_23_P45999 | FBXO2 | 1p36.22 | 0.02707 | 0.00124 | 0.18299 |
| A_23_P144384 | GALNT7 | 4q34.1 | 0.02462 | 0.00149 | 0.13454 |
| A_23_P327777 | GATS | 7q22.1 | 0.009291 | 0.00003 | -0.53148 |
| A_24_P230502 | HSD17B1 | 17q21.31 | 0.001512579 | 0.112782075 | 0.238579582 |
| A_24_P161525 | HSP90AB3P | 4q22.1 | 0.571945186 | 0.006544174 | -0.073205595 |
| A_24_P372012 | ICA1 | 7p21.3 | 0.009883 | <0.000001 | -0.18 |
| A_24_P20795 | IRX4 | 5p15.33 | 0.002325267 | 0.341617432 | 0.060042723 |
| A_23_P381992 | ITGAV | 2q32.1 | 0.045298 | 0.00004 | -0.58283 |
| A_23_P113462 | KIF21A | 12q12 | 0.021206 | <0.000001 | -0.54318 |
| A_24_P792748 | KRT8P17 | Xp11.1 | 0.020131 | <0.000001 | -0.56552 |
| A_24_P7021 | KRT8P19 | 12q14.1 | 0.01182 | 0.00032 | -0.74858 |
| A_23_P301995 | LIN9 | 1q42.12 | 0.002395 | 0.00457 | 0.0752 |
| A_24_P381441 | LMO3 | 12p12.3 | 0.10084 | <0.000001 | 0.20291 |
| A_24_P16071 | LOC442256 | 6q22.32 | 0.043289 | 0.00031 | -0.63556 |
| A_24_P341603 | LOC643594 | 5p15.33 | 0.001775947 | 0.137817896 | 0.317632268 |
| A_24_P144337 | LOC651417 | 2p23.3 | 0.005076 | <0.000001 | -0.55451 |
| A_24_P324284 | LOC728550 | 1p36.33 | 0.000692 | 0.18187 | 0.29487 |
| A_24_P148653 | LSG1 | 3q29 | 0.018487 | 0.00003 | -0.12674 |
| A_24_P10731 | MADCAM1 | 19p13.3 | 0.005316 | <0.000001 | 0.29738 |
| A_24_P117301 | MCM3APAS | 21q22.3 | 0.00189639 | 0.274005598 | 0.055221169 |
| A_24_P314640 | MDGA1 | 6p21.2 | 0.039224 | <0.000001 | 0.25067 |
| **Probe ID** | **Gene Symbol** | **Cytoband** | **P kruskal^1^** | **P linear^2^** | **Slope^3^** |
| A_24_P241370 | MTA1 | 14q32.33 | 0.000534 | 0.26551 | 0.02995 |
| A_32_P17182 | N48043 | 15q14 | 0.044554 | <0.000001 | 0.34181 |
| A_23_P1740 | NCAM1 | 11q23.1 | 0.47237 | NA | NA |
| A_23_P10463 | NDUFS5 | 1p34.3 | 0.24653 | 0.00075 | -0.05771 |
| A_24_P129107 | NKD2 | 5p15.33 | 0.000697 | 0.19526 | 0.39962 |
| A_24_P406601 | OLFM1 | 9q34.3 | 0.039342 | <0.000001 | -0.97234 |
| A_24_P314337 | PAGE1 | Xp11.23 | NA | NA | NA |
| A_23_P134384 | PHF14 | 7p21.3 | 0.018104 | <0.000001 | -0.40575 |
| A_24_P186030 | PRKY | Yp11.2 | 0.039164 | 0.00049 | -0.40726 |
| A_24_P535483 | PRR17 | 20q13.33 | 0.00397268 | 0.348990225 | 0.325576983 |
| A_24_P192262 | RALA | 7p14.1 | 0.008781 | 0.00018 | -0.18771 |
| A_24_P824592 | RBMX | Xq26.3 | 0.012811 | <0.000001 | -0.54303 |
| A_23_P26468 | RHBDL1 | 16p13.3 | 0.000208 | 0.17758 | 0.39905 |
| A_23_P55616 | SLC14A1 | 18q12.3 | 0.018535 | 0.00038 | 0.57681 |
| A_23_P358548 | SLC43A3 | 11q12.1 | 0.63474 | 0.00088 | 0.20624 |
| A_24_P65292 | SOX8 | 16p13.3 | 0.003189116 | 0.286223574 | 0.352186718 |
| A_24_P322771 | TFF1 | 21q22.3 | 0.003497588 | 0.014039977 | 0.091260293 |
| A_23_P393099 | TFF3 | 21q22.3 | 0.341068466 | 0.004269515 | 0.262026111 |
| A_24_P313576 | VAMP2 | 17p13.1 | 0.001654649 | 0.105618295 | 0.047878583 |
| A_24_P129326 | VGF | 7q22.1 | 0.009682 | <0.000001 | 0.24364 |
| A_24_P282261 | ZNF493 | 19p12 | 0.000869 | <0.000001 | -0.63901 |

^1^Non-parametric Kruskall-Wallis test for differential gene expression across three dose categories (≤ 0.30, 0.31-1.0, > 1.0 Gy).^2^Linear trend test for differential gene expression with continuous dose. ^3^Estimate of linear dose-response slope based on continuous dose.
