# Supplementary material for: Iodine-131 Dose Dependent Gene Expression in Thyroid Cancers and Corresponding Normal Tissues Following the Chernobyl Accident
Source: PLoS One. 2012 Jul 25;7(7):e39103. doi: 10.1371/journal.pone.0039103 (PMC3405097; doi:10.1371/journal.pone.0039103)
Supplement: Table S2 — Summary information for 11 genes with significant differential dose-expression relationship based on qRT-PCR measurements. Note: 1Gene description and mechanism of action as defined in National Center for Biotechnology Information (NCBI) Entrez Gene, June 2011. 2Based on review of Gene References Into Functions (RIF) and NCBI bibliography as well as additional literature searches in PubMed focused on reports of thyroid cancer or cancer-related biological processes. Abbreviations: TGF-beta, transforming growth factor-beta; PTC, papillary thyroid cancer; ALL, acute lymphoblastic anemia. (DOC) [file pone.0039103.s005.doc]

Supplemental Table 2. Summary information for 11 genes with significant dose-expression relationship based on qRT-PCR measurements.

| **Gene (Gene ID)** | | **Cytoband** | **Gene’s function^1^** | **Gene’s mechanism^1^** | | **Gene RIF**^2^ **and bibliography** | **Selected references** |
| --- | --- | --- | --- | --- | --- | --- | --- |
| ***Cell cycle & growth and differentiation*** | | | |  | |  |  |
| Cyclin-dependent kinase 12 (*CDK12*) | | 17q12 | Protein kinase involved in cell cycle or transcription | Association with specific cyclin subunits for e.g. cell cycle progression | | CDK12 is a newly proposed family member which name is based on the presence of a cyclin-binding element | Malumbres et al., Nat Cell Biol. 2009 Nov;11(11):1275-6 |
|  |  |  |  |  |  | CDK12 silencing causes loss of estrogene receptor dependence and resistance to therapy for breast cancer | Iorns et al., Carcinogenesis. 2009 Oct;30(10):1696-701 |
| Activin A receptor, type IIA (*ACVR2A*) | | 2q22.3 | Activins are growth and differentiation factors, belonging to the TGF-beta superfamily and are considered to be constitutive active kinases | Type I and II receptors form a stable complex after ligand binding which result in phosphorylation of type I receptors by type II receptors | | Mutation in activin type II receptor is associated with colorectal cancer | Campregher et al., Clin Cancer Res. 2010 Mar 15;16(6):1950-6 |
|  |  |  |  |  |  | *ACVR2* gene is mutated in 92% of colorectal cancers with microsatellite instability | Tougeron et al., Mod Pathol. 2009 Sep;22(9):1186-95 |
|  |  |  |  |  |  | Activin receptor signaling regulates prostatic epithelial cell adhesion and viability | Simon et al., Neoplasia. 2009 Apr;11(4):365-76 |
| ***Cell adhesion*** | | | |  | |  |  |
| Adherens junctions associated protein 1 (*AJAP1*) | | 1p36.32 | Influences cellular invasion and migration through adherens junctions | AJAP1 is a membrane protein which interacts with E-cadherin-catenin complexes of adherens junctions | | AJAP1 influences cell invasion and interacts with invasion-promoting protein CD147 | Schreiner et al., Mol Biol Cell. 2007 Apr;18(4):1272-81 |
|  |  |  |  |  |  | *AJAP1* gene, frequently deleted in oligodendrogliomas, functions to inhibit cell adhesion and migration | McDonald et al., Cancer Biol Ther. 2006 Mar;5(3):300-4 |
| Family with sequence similarity 38, member A (*FAM38A*) | | 16q24.3 | Encodes PIEZO1, a protein that induces mechanically activated currents in various cell types | Large transmembrane proteins conserved among various species, all having between 24 and 36 predicted transmembrane domains | | *FAM38A* siRNA knockdown in epithelial cells inactivates endogenous beta1 integrin, reducing cell adhesion | McHugh et al., J Cell Sci. 2010 Jan 1;123(Pt 1):51-61 |
|  | | | | | | | |
|  | | | | | | | |
|  | | | | | | | |
| ***Microenvironment/metabolic changes (e.g. acid, energy metabolism)*** | | | | | | | |
| Carbonic anhydrase  (*CA12*) | 15q22.2 | | Influences acid-base balance in cerebrospinal fluid, saliva, and stomach. It is a type I membrane protein highly expressed in normal tissues, such as kidney, colon, and pancreas | Zinc metalloenzyme that catalyze the reversible hydration of carbon dioxide | Potential marker of radiation-related PTC | | Stein et al., Thyroid. 2010 May;20(5):475-87 |
|  |  |  |  |  | Potential marker of follicular thyroid tumors | | Fryknäs et al., Tumour Biol. 2006;27(4):211-20 |
|  |  |  |  |  | *CA12* expression is associated with histologic grade of cervical cancer and radiotherapy outcome | | Nam et al., Radiat Oncol. 2010 Nov 1;5:101 |
|  |  |  |  |  | *CA12* expression promotes invasion and migration of breast cancer cells through p38 MAPK | | Hsieh et al, Eur J Cell Biol. 2010 Aug;89(8):598-606 |
|  |  |  |  |  | *CA 12* expression predicts poor prognosis in meduloblastoma and neuroectodermal tumors | | Nordfors et al., BMC Cancer. 2010 Apr 18;10:148 |
|  |  |  |  |  | CA 12 protein participates in pH regulation, which is important for survival of hypoxic cancer cells | | Pastorekova et al., Curr Pharm Des. 2008;14(7):685-98 |
| ***Transcription factor and DNA modification through methylation*** | | | |  |  | |  |
| LIM domain only 3, rhombotin-like 2 (*LMO3*) | | 12p12.3 | Encodes a cysteine-rich transcriptional regulator | Might act as a co-repressor of p53 and the neuronal transcription factor HEN2 | High expression contributes  to the development and aggressiveness  of neuroblastoma | | Larsen et al., Biochem Biophys Res Commun. 2010 Feb 12;392(3):252-7 |
|  |  |  |  |  | Interacts with neuronal transcription factor, HEN2, and acts as an oncogene in neuroblastoma | | Aoyama et al., Cancer Res. 2005 Jun 1;65(11):4587-97 |
| Zinc finger protein 493 (*ZNF493*) | | 19p12 | Regulates transcription, DNA-dependent | DNA-binding, zinc-binding | No association with cancer is described | |  |
| Metastasis associated 1 (*MTA1*) | | 14q32.33 | Contains a dimerization domain and a domain commonly found in proteins that methylate DNA | Involved in regulating transcription which may be accomplished by chromatin remodeling | Plays a role in repair of double strand breaks caused by ionizing radiation and the UV-induced ATR-mediated DNA damage checkpoint pathway | | Li et al., J Biol Chem. 2010 Jun 25;285(26):19802-12 |
|  |  |  |  |  | Part of the Mi-2/NuRD complex containing e.g. a histone deacetylase; probably functions in transcriptional repression | | Bowen et al., Biochim Biophys Acta. 2004 Mar 15;1677(1-3):52-7 |
|  |  |  |  |  | *MTA1* expression is an independent prognostic factor for patients with non-small-cell lung cancer | | Yu et al., Interact Cardiovasc Thorac Surg. 2011 Feb;12(2):166-9 |
|  |  |  |  |  | Pro-angiogenic and pro-invasive functions create permissive environment for prostate tumor growth and likely to support metastasis | | Kai et al., Prostate. 2011 Feb 15;71(3):268-80 |
|  |  |  |  |  | MTA1 expression is associated with invasive ductal breast carcinoma | | Sharma et al., Tumour Biol. 2011 Feb;32(1):23-32 |
|  |  |  |  |  | Induces *VEGF-C* expression and facilitates lymphangiogenesis in colorectal cancer | | Du et al., World J Gastroenterol. 2011 Mar 7;17(9):1219-26 |
| Solute carrier family 19, (folate transporter), member 1 (*SLC19A1*) | | 21q22.3 | Membrane protein regulating intracellular folate concentration | Transporter of folate; folates maintain DNA stability through their ability to donate one-carbon units (methyl-group) for cellular metabolism | Polymorphisms in *SLC19A1* gene are associated with non-small cell lung cancer | | Adjei et al., J Thorac Oncol. 2010 Sep;5(9):1346-53 |
|  |  |  |  |  | Folate pathway is important in the maintenance of genomic stability; its disregulation has been linked with childhood ALL | | Chan et al., Hematol Oncol. 2010 Sep 7. [Epub ahead of print] |
|  |  |  |  |  | Folate is required for methylation of e.g. cytosine and is needed for  control of gene expression | | Fenech, Mech Ageing Dev. 2010 Apr;131(4):236-41 |
|  |  |  |  |  | Folate deficiency is linked with several cancers, e.g. colorectum, breast, ovary, pancreas, brain, lung, and cervix | | Duthie, J Inherit Metab Dis. 2011 Feb;34(1):101-9 |
| ***Unknown function or poorly characterized*** | | | |  |  | |  |
| UDP-N-acetyl-alpha-D-galactosamine:polypeptide N-acetylgalactosaminyltransferase 7  (*GALNT7*) | | 4q31.1 | Member of the GalNAc-transferase family | Controls mucin-type O-linked protein glycosylation and transfer of N-acetylgalactosamine to serine and threonine amino acid residues | No association with cancer is described | |  |
| Solute carrier family 43, member 3 (*SLC43A3*) | | 11q11 | Member of solute carrier (SLC)-class of transport proteins | Transmembrane transport | Involved in transport of nutrients in rapidly growing and/or developing tissues | | Stuart et al., Am J Physiol Renal Physiol. 2001 Dec;281(6):F1148-56 |

^1^Gene description and mechanism of action as defined in NCBI Entrez Gene, June 2011. ^2^Based on review of Gene References Into Functions (RIF) and NCBI bibliography as well as additional literature searches in PubMed focusing on reports of thyroid cancer or cancer-related biological processes. Abbreviations: TGF-beta, transforming growth factor-beta; PTC, papillary thyroid cancer; ALL, acute lymphoblastic anemia.
